# Light and compressed gluinos at the LHC via string theory

**DOI:** 10.1140/epjc/s10052-017-4891-9

**Published:** 2017-05-16

**Authors:** S. S. AbdusSalam

**Affiliations:** 1 Department of Physics, Shahid Beheshti University, Tehran, 19839 Islamic Republic of Iran; 2 INFN, Sezione di Roma, c/o Department of Physics, University of Rome “La Sapienza”, Rome, Italy

## Abstract

In this article, we show that making global fits of string theory model parameters to data is an interesting mechanism for probing, mapping and forecasting connections of the theory to real world physics. We considered a large volume scenario (LVS) with D3-brane matter fields and supersymmetry breaking. A global fit of the parameters to low-energy data shows that the set of LVS models are associated with light gluinos which are quasi-degenerate with the neutralinos and charginos they can promptly decay into, and thus they are possibly hidden to current LHC gluino search strategies.

## Introduction

Flux compactification with stabilised moduli sets a connection of string theory models to real world physics [1, 2]. The parameters describing the various sets of string theory models lead to different physical properties. This is a feature associated to the so-called “landscape” phenomenon of string theory vacua. The KKLT [3, 4] and large volume scenarios [5, 6] are famous examples with each representing sets of string theory models. Within each of these, there are additional model variations including differences in gauge groups, particle representations and the cosmological constants for representing the physical world. Our target in this article is relating the sets of model predictions to collider observables and, eventually, to collider phenomenology.

The vacuum that we inhabit should be just one from the set of landscape vacuum possibilities. An interesting question goes as follows. Is it possible to have an explicit set of string theory-based models which is in complete agreement with our real world? Here real world can be taken to mean the standard models of particle physics and cosmology, or even including their extensions such as those with supersymmetry breaking. For the latter case, if one assumes that supersymmetry exists in nature but is broken at some energy scale accessible to colliders, then predictions for supersymmetry breaking from flux compactification models can be used for assessing the string theory model parameters.^1^ This is also true for cosmological observables that can be related to moduli fields. But, in this article, the focus is on the particle physics aspect.

Sampling string theory-based model parameters for making global fits to certain experimental data, representing the real world, could be the best way for finding explicit models in agreement with observations. For this purpose, here, the Bayesian techniques for fitting models to data [10, 11] will be employed and applied to the string theory models. Based on the experimental results used, statistical weights will be assigned to points for mapping and making forecasts as regards the parameter space. This approach is new within string theory phenomenological research (see [12] for an overview).

For instance, consider a IIB string theory model with the dilaton and complex structure moduli stabilised via the flux superpotential *W* [13, 14]. Its vacuum expectation value (VEV), $W_0$, affects the physical properties of the string vacuum, such as the magnitude of the cosmological constant and scale of supersymmetry breaking. A scan over the landscape of flux compactifications can be achieved by varying the value of the flux superpotential $W_0$. In [15], by considering a particular Calabi–Yau manifold with two moduli fields and starting from the large volume scenario (LVS) [5, 6] limit, with $W_0 \sim 1$, or the KKLT limit, with $W_0 \ll 1$ [3, 4], a set of flux-dependent AdS and dS vacua (without uplift terms) were obtained. Some of the minima have supersymmetry spontaneously broken by the fluxes for matter fields on D7 branes. One can go further by computing the full-fledged supersymmetry spectrum via RG-running of the predicted supersymmetry-breaking terms to the electroweak scale such as in [16]. Given the supersymmetry spectrum, other observables, such as the Higgs boson mass, dark matter relic density, electroweak precision and B-physics observables, can be computed. Non-agreement between the predicted observables and their corresponding experimentally measured values or limits can be used for marking the point $W_0$ as not suitable for representing our world. This way, statistical methods for exploration and fitting the landscape parameters can be used for mapping and physics forecasting.

In this article, we consider a particular LVS model [20] with matter fields on a D3-brane and supersymmetry broken via a chiral superfield *X* with nilpotent constraint ($X^2=0$) [17–19]. In Sect. 2 we briefly introduce the LVS and the supersymmetry-breaking terms derived in [20] for setting the context of the article and defining an LVS subset of the minimal supersymmetric standard model (MSSM), with R-parity conserved, called LMSSM-6, as the phenomenological frame for our analysis. The Bayesian method for fitting LMSSM-6 to low-energy physics is presented in Sect. 3. The results of the global fit indicate an abundance of light gluinos, neutralinos and charginos together with $\mathcal{O}(10)$ TeV squarks. The electroweak inos have one to few top-quark masses. The mass difference between the gluinos, charginos or heavy neutralinos and the lightest supersymmetric particle (LSP) turns out to be small, thereby making a lower sub-TeV quasi-degenerate spectrum. As such the gluinos and electroweak inos are possibly surviving current LHC limits. Detailed reinterpretations or recasting analyses should ultimately lead to a robust conclusion as regards the status of such a spectrum at the LHC. In Sect. 4 we finalise the article with conclusions and an outlook.

## Large volume scenario (LVS) SUSY-breaking, LMSSM-6

The shape and volume of string theory’s internal space represent massless scalar fields from a 4-dimensional theory point of view. These moduli fields must be stabilised since no such extra scalar fields are observed in nature. There is much work on moduli stabilisation in the literature. Here we concentrate on an LVS scenario where all the moduli fields are fixed at an exponentially large internal volume. The LVS supergravity effective theory couples to the particle content of the MSSM. In the effective theory, the Kähler and superpotentials are generated from the superstring theory. This forms the hidden sector, which then couples via gravity mediation to the MSSM sector. 4d $N=1$ supergravity is specified up to two derivatives by the Kähler potential *K*, the superpotential *W* and the gauge kinetic function $f_a$. With these, the scalar potential is given by

1 $$\begin{aligned}&V = e^K \left[ G^{i\bar{j}} D_i W \overline{D_j W} - 3|W|^2\right] , \end{aligned}$$

2 $$\begin{aligned}&D_i W = \partial _i W + (\partial _i K)W,\end{aligned}$$

3 $$\begin{aligned}&G^{i\bar{j}} = (\partial _i \partial _{\bar{j}} K)^{-1}. \end{aligned}$$

Here *i*, *j* run over the two Kähler moduli $T_s$ and $T_b$ whose real parts $\tau _b$ and $\tau _s$ determine the internal space volume, $\mathcal {V} = \tau _b^{3/2}-\tau _s^{3/2}$. At the minimum of the potential the moduli fields acquire VEVs and provide a non-vanishing auxiliary field that spontaneously breaks supersymmetry and generate the breaking terms in the visible sector. Other dynamics such as the presence of D3-brane within the superstring construction can also lead to the breaking of supersymmetry.

The LVS supersymmetry-breaking terms induced by the presence of the nilpotent superfield *X* [20] is presented as follows. The Kähler potential and superpotentials for *X* can be taken as

4 $$\begin{aligned} K\!=\!K_0 + K_1X + \bar{K}_1\bar{X}\ + K_2X\bar{X} , \quad W=\rho X + W_0, \end{aligned}$$

where $K_0, K_1, K_2, \rho , W_0$ are coefficients and can be functions of other low-energy fields. Assuming the dilaton and complex structure moduli have been fixed and integrated out at high scale, the LVS Kähler potential becomes

5 $$\begin{aligned} K = -2\log \left( \mathcal {V}- \hat{\xi }\right) + \tilde{K}_i\ \phi \bar{\phi } + \tilde{Z}_i\ X \bar{X} + \tilde{H}_i\ \phi \bar{\phi } \ X \bar{X} + \cdots . \end{aligned}$$

**Table 1:** Summary for the central values and errors for the electroweak precision, B-physics and cold dark matter relic density constraints

Observable	Constraint	Observable	Constraint
$m_W$ [GeV]	$80.399 \pm 0.023$ [22]	$A^l = A^e$	$0.1513 \pm 0.0021$ [23]
$\Gamma _Z$ [GeV]	$2.4952 \pm 0.0023$ [23]	$A^b$	$0.923 \pm 0.020$ [23]
$\sin ^2\, \theta _\mathrm{eff}^{lep}$	$0.2324 \pm 0.0012$ [23]	$A^c$	$0.670 \pm 0.027$ [23]
$R_l^0$	$20.767 \pm 0.025$ [23]	$\mathrm{BR}(B_s \rightarrow \mu ^+ \mu ^-)$	$3.2^{+1.5}_{-1.2} \times 10^{-9}$ [24]
$R_b^0$	$0.21629 \pm 0.00066$ [23]	$\Delta M_{B_s}$	$17.77 \pm 0.12$ ps$^{-1}$ [25]
$R_c^0$	$0.1721 \pm 0.0030$ [23]	$R_{\mathrm{BR}(B_u \rightarrow \tau \nu )}$	$1.49 \pm 0.3091$ [26]
$A_{\text {FB}}^b$	$0.0992 \pm 0.0016$ [23]	$\Delta M_{B_d}$	$0.507 \pm 0.005$ ps$^{-1}$ [27]
$A_{\text {FB}}^c$	$0.0707 \pm 0.0035$ [23]	$\Omega _{CDM} h^2$	$0.11 \pm 0.02$ [28]
$m_h$ [GeV]	$125.6 \pm 3.0$ [29, 30]	$\mathrm{BR}(B\rightarrow X_s \gamma )$	$(3.52 \pm 0.25) \times 10^{-4}$ [31]

Here $\hat{\xi }= \frac{s^{3/2}}{\xi }{2}$, $\xi$ is a Calabi–Yau manifold related constant of order one [21]. $s=1/g_s$ is the real part of the axion–dilaton field. $\phi$ represents matter, $\tilde{K}_i$ and $\tilde{Z}_i$ are, respectively, the D3-brane matter and the nilpotent goldstino metrics. $\tilde{H}_i$ represents the nilpotent goldstino and the matter field quartic interaction. These are parameterised for the $\alpha ^{'}$-corrected potential as

6 $$\begin{aligned}&\tilde{K}_i \!=\! \frac{\alpha _0}{\mathcal {V}^{2/3}}\left( 1-\alpha _1 \frac{\xi s^{3/2}}{\mathcal {V}} \right) , \quad \tilde{Z}_i = \frac{\beta _0}{\mathcal {V}^{2/3}}\left( 1-\beta _1 \frac{\xi s^{3/2}}{\mathcal {V}} \right) , \nonumber \\&\tilde{H}_i = \frac{\gamma _0}{\mathcal {V}^{4/3}}\left( 1-\gamma _1 \frac{\xi s^{3/2}}{\mathcal {V}} \right) . \end{aligned}$$

The LVS superpotential is given by

7 $$\begin{aligned} W = W_0 + \rho X + A e^{-a_s T_s}, \end{aligned}$$

where *A*, and $a_s$ are gaugino condensation parameters. The Kähler and superpotentials can then be used for computing the soft supersymmetry-breaking terms via a standard method as done in [20]. The scalar $m_0$, gaugino $M_{1/2}$, and trilinear coupling $A_0$ soft supersymmetry-breaking terms for visible sector fields on the D3-brane are

8 $$\begin{aligned} m_0^2= & {} \frac{5}{4} \frac{s^{3/2} \xi }{\mathcal V} \, (3 \alpha _1 -1)\, m_{3/2}^2 \nonumber \\&+ \frac{9}{8}\frac{s^{3/2} \, \xi }{\mathcal {V}} \, \frac{1}{5a_s \tau _s} \, \left( 1-\frac{3 \gamma _0}{\alpha _0 \beta _0} \right) m_{3/2}^2, \end{aligned}$$

9 $$\begin{aligned} M_{1/2} = \mathrm{sign}(W_0) \, \frac{3}{4}\frac{s^{3/2}\xi }{\mathcal V} \left[ 3 - 2\omega _s \right] \,m_{3/2}, \text { and } \end{aligned}$$

10 $$\begin{aligned} A_0 = - (1- y) M_{1/2} , y = s\partial _s \log Y_{ijk}^{(0)}. \end{aligned}$$

Here $m_{3/2} = e^{K/2} |W|$ is the gravitino mass. $\omega _s \lesssim 1$ parametrises corrections such that $D_sW \sim \frac{e^{-a\,\tau }}{2s}\omega _s$ as used in computing the soft supersymmetry-breaking terms.

Given a set of LMSSM-6 parameters, the supersymmetry-breaking terms Eqs. (8)–(10) can be computed. These are then set as the boundary conditions for renormalisation group (RG) running from the supersymmetry-breaking scale, $m_{3/2}$, to the weak scale. For doing this we chose a particular specialisation of gravity-mediated supersymmetry breaking with a non-universal scalar mass terms for the MSSM Higgs doublets such that $m_{H_1} = m_{H_2} = 0$ at symmetry-breaking scale. Other possibilities include varying the non-universal Higgs doublet mass terms and the minimal supergravity but are not considered here. The set of LVS parameters, $\{ \, x, \, \alpha _1, \, y, \, \omega _s, \, \tan \beta , \, \log _{10} m_{3/2} \}$, in the soft supersymmetry-breaking terms above together the RG to the weak scale with MSSM sparticle content is referred to as the LVS MSSM with six parameters (LMSSM-6). $\tan \beta$ is the ratio of the Higgs doublet VEVs. For making a global fit of the LMSSM-6 parameters to data we add five standard model nuisance parameters that are used for some of the precision observables. As such we will be exploring a total of 11 parameters,

11 $$\begin{aligned}&\underline{\theta } \equiv \left\{ \, x, \, \alpha _1, \, y, \, \omega _s, \, \tan \beta , \, \right. \nonumber \\&\qquad \quad \left. \log _{10} m_{3/2}, m_Z, \, m_t, \, m_b, \, \alpha _{em},\, \alpha _s \right\} . \end{aligned}$$

The standard model nuisance parameters are the Z-boson mass $m_Z = 91.1876 \pm 0.0021$ GeV, the top-quark mass $m_t = 172.6 \pm 1.4$ GeV, the bottom quark mass $m_b = 4.2 \pm 0.07$ GeV, the electromagnetic coupling constant $\alpha _{em}^{-1} = 127.918 \pm 0.018$, and the strong interaction coupling constant $\alpha _s = 0.1172 \pm 0.002$. These were all set to vary in a Gaussian manner with central values and deviations according to the experimental results. The LVS parameters were allowed to vary as $x \equiv \frac{3 \gamma _0}{\alpha _0 \beta _0} \in [0.01, 100.0]$, $\alpha _1 \in [0.01, 100.0]$, $y \in [-1000, 1000]$, $\omega _s \in [0.01, 100.0]$, $\tan \beta \in [2, 60]$, $\log _{10} m_{3/2} \in [3, 19]$. The other LVS parameters which were not varied but enter the soft terms are $\xi =1.0, W_0=\pm 1.0,s=25.0$, and ${\mathcal V}=1.0 \times 10^{9}$ units. In the next section we describe the procedure for fitting the parameters to low-energy data.

**Fig. 1:**
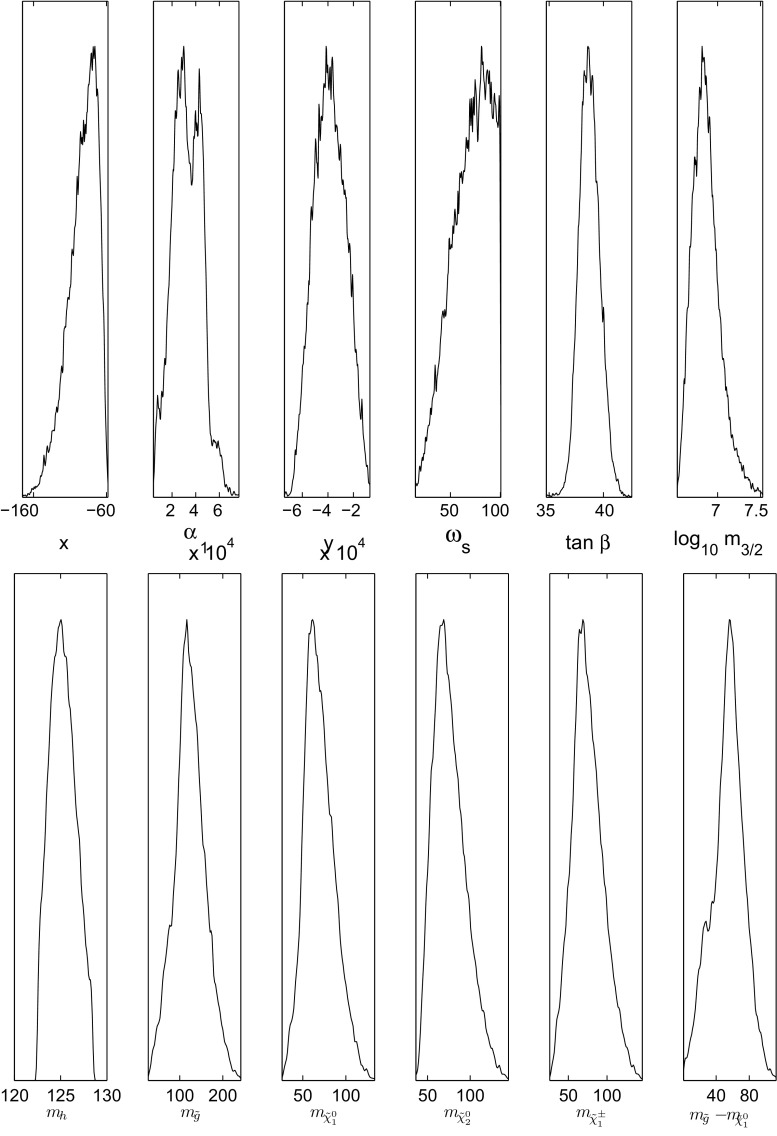
*First row* One-dimensional posterior probability distributions for the SUSY-breaking parameters and (*second row*) for the Higgs boson and electroweak ino mass in GeV units. Corresponding squark masses (not shown here) are all of the order of 10 TeV. The *vertical axis* is a measure of the relative posterior probabilities between the *x*-axis points

## The LMSSM-6 fit to low-energy physics

We consider a flat (Gaussian) prior probability density distribution, $p(\underline{\theta })$, for the LMSSM-6 (standard model) parameters in Eq. (11) for exploring the space using the MultiNest [32, 33] package which implements the nested sampling algorithm [34]. During the model exploration, at each parameter-space point the following packages were used via the SLHA1 [35] interface: the supersymmetry spectrum generator SOFTSUSY [36] for computing the sparticle masses, mixing angles and couplings, micrOMEGAs [37] for computing the neutralino cold dark matter relic density and the anomalous magnetic moment of the muon $\delta a_\mu$, SuperIso [38] for predicting the branching ratios $\mathrm{BR}(B_s \rightarrow \mu ^+ \mu ^-)$, $\mathrm{BR}(B \rightarrow s \gamma )$ and the isospin asymmetry, $\Delta _{0-}$ in the decay $B \rightarrow K^* \gamma$, and susyPOPE [39, 40] for computing precision observables that include the *W*-boson mass $m_W$, the effective leptonic mixing angle variable $\sin ^2 \theta ^{lep}_\mathrm{eff}$, the total *Z*-boson decay width, $\Gamma _Z$, and the other electroweak observables whose experimentally determined central values and associated errors are summarised in Table 1. The experimental central values ($\mu _i$) and errors ($\sigma _i$) for these make the set of the predictable observables, $\underline{O}$:

12 $$\begin{aligned} \underline{O}\equiv & {} \left\{ m_h, \; m_W,\; \Gamma _Z,\; \sin ^2\, \theta ^{lep}_\mathrm{eff},\; R_l^0,\; R_{b,c}^0,\; A_{FB}^{b,c},\; A^l = A^e,\; A^{b,c}, \right. \nonumber \\&\left. \mathrm{BR}(B \rightarrow X_s \, \gamma ),\; \mathrm{BR}(B_s \rightarrow \mu ^+ \, \mu ^-),\; \Delta M_{B_s},\; R_{\mathrm{BR}(B_u \rightarrow \tau \nu )},\right. \nonumber \\&\left. \Omega _{CDM}h^2, \; \mathrm{BR}(B_d \rightarrow \mu ^+ \mu ^-), \; \Delta M_{B_d} \right\} . \end{aligned}$$

The compatibility of the LMSSM-6 parameter-space points with the data is quantified by the likelihood, $p(\underline{d}|\underline{\theta })$. Assuming the observables are independent, the combined likelihood can calculated as

13 $$\begin{aligned} p(\underline{d}|\underline{\theta }) = L(x) \, \prod _i \, \frac{ \exp \left[ - (O_i - \mu _i)^2/2 \sigma _i^2\right] }{\sqrt{2\pi \sigma _i^2}}, \end{aligned}$$

where the index *i* runs over the list of observables $\underline{O}$. Here, *x* represents the predicted value of neutralino cold dark matter (CDM) relic density and

14 $$\begin{aligned} L(x) = {\left\{ \begin{array}{ll} 1/(y + \sqrt{\pi s^2/2}) &{}\quad \text {if } x < y \\ \exp \left[ -(x-y)^2/2s^2\right] /(y + \sqrt{\pi s^2/2}) &{}\quad \text {if } x \ge y \end{array}\right. }, \end{aligned}$$

where $y = 0.11$ is the CDM relic density central value and $s=0.02$ the corresponding inflated error (to allow for theoretical uncertainties).

**Fig. 2:**
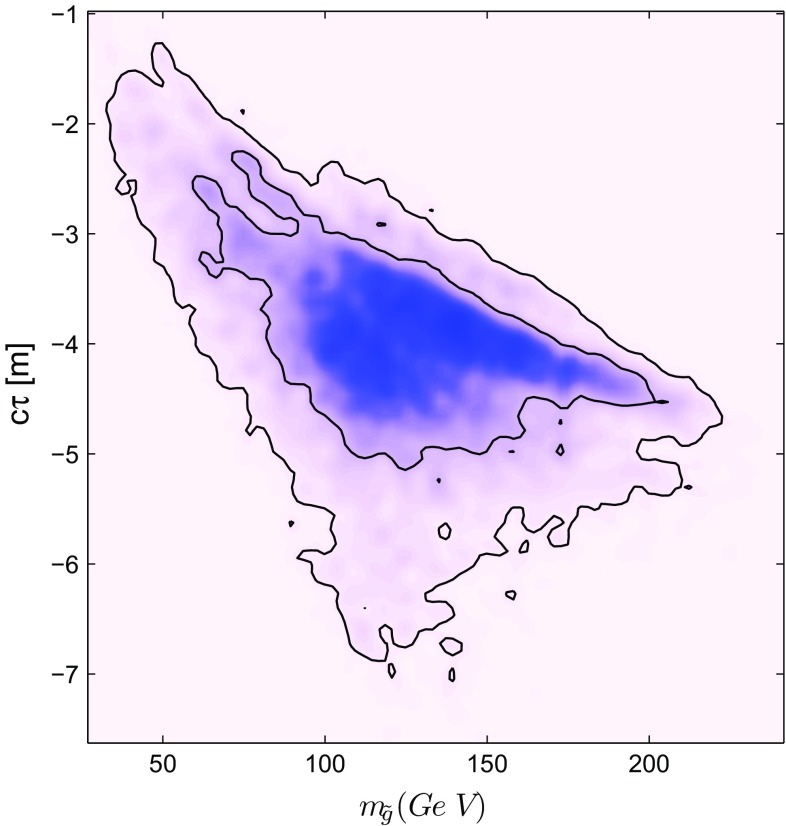
Marginalised 2-dimensional posterior distribution for the gluino decay length versus the gluino mass. The *contour lines* show the 68 and 95% Bayesian credibility regions

The outcome of the Bayesian fit of the LMSSM-6 to data is the posterior probability density

15 $$\begin{aligned} p(\underline{\theta }|\underline{d}) = \frac{p(\underline{d}|\underline{\theta }) \, \times \, p(\underline{\theta })}{p(\underline{d})}, \end{aligned}$$

which shows that the sfermions have masses of the order of 10 TeV, while the gluino and electroweak inos are light, with masses in the range of a few hundred GeV. The posterior probability distributions for the LMSSM-6 supersymmetry-breaking parameters, the Higgs boson mass for reference purpose, the electroweak inos and the gluino–neutralino mass difference are shown in Fig. 1. The heavy squarks can potentially make the gluinos long-lived or make them turn into R-hadrons before decaying. In Fig. 2, the 2-dimensional posterior distribution for the gluino decay length estimate versus its mass is given. The gluino decay life-time for the squark at the order of 10 TeV is estimated as [41, 42]

16 $$\begin{aligned} \tau \approx 8 \, \left( \frac{\tilde{m}}{10^9 \, \text { GeV} }\right) ^4 \, \left( \frac{ 1 \, \text { TeV} }{m_{ \tilde{g}} }\right) ^5 \,\, \mathrm{s}. \end{aligned}$$

It turns out that the gluino and electroweak inos are compressed relative to the LSP mass. For this reason, one cannot automatically rule out LMSSM-6 light gluinos.

The direct search for gluinos at the LHC puts the lower limit on the gluino mass well into the TeV towards multi-TeV region (see for instance Refs. [43–45]). The limits come from search channels with multijets plus zero leptons and can probe gluinos decaying into gluons and neutralinos. The same is the case for the gluino decay to two quarks and a neutralino; and also for the topologies with initial state radiations. However, it is not clear what the status will be for the light gluinos which are quasi-degenerate with the neutralinos or charginos they can decay to. The gluino production cross section at the LHC is much higher than the squarks’ of the same mass. So in principle as long as the gluino decays to a gluon and neutralino or to a quark pair and neutralino, then, for instance, the CMS limits from searches with multijet final states [44, 45] should apply for the LMSSM-6 gluinos. But this and the application of other relevant LHC limits necessarily require a rigorous and dedicated reinterpretation analysis of the results.

In [46] various experimental search strategies for finding gluinos quasi-degenerate with neutralinos have been addressed. These include searches for displaced vertices and disappearing tracks. The analysis was, however, based on a fixed gluino mass at 1.5 TeV. It will be interesting to see what the outcome of a similar analysis will be for light gluinos such as for the LMSSM-6 shown in this article. We again emphasise that a decisive conclusion requires a detailed reinterpretation of the experimental results within the LMSSM-6 context.

## Conclusion and outlook

Models from string theory compactification with moduli stabilised by fluxes can predict supersymmetry breaking at the TeV scale. The generation of supersymmetry spectrum allows the connection of the string theory or landscape parameters to experimental observables for constraining the theory. In this article, we have applied the Bayesian global fit technique for string theory inspired phenomenology. We introduce LMSSM-6 for representing the set of supersymmetry-breaking parameters predicted from a large volume scenario with matter fields on D3-branes. The field contents was considered to be that of the minimal supersymmetric standard model with R-parity conserved and the neutralino as the lightest supersymmetric particle as dark matter candidate. This way, the LMSSM-6 parameters which originate from the string theory setting can be constrained via the low-energy properties of the associated sparticle spectrum. This feature was used for forecasting and mapping the string theory-based parameter space.

The low-energy constraints used are the Higgs boson mass, dark matter relic density, electroweak precision and B-physics experimentally measured observables. The global fit of the LMSSM-6 parameters to these showed that the bulk of the posterior distribution yielded heavy squarks at order 10 TeV. These come together with light (much less than or of the order of the top-quark mass) but promptly decaying gluinos. Such gluinos would have been already ruled out even before the LHC if not for being quasi-degenerate with neutralinos and charginos. A dedicated reinterpretation of the experimental results that probe gluino productions at the LHC, such as in [43–45], is needed for determining the status of the gluinos. This is an interesting issue, but it is beyond the scope of this article. Should the LMSSM-6 gluinos be ruled out, then the corresponding class of large volume scenario models cannot represent our real world.

As an outlook, the following are interesting issues based on the concepts addressed in this article.

- The methodology presented can be applied to other string theory phenomenology frames such as the famous KKLT [3, 4] scenarios in comparison to the large volume ones.
- The posterior sample from the global fit of parameters to data can be used in recasting collider results for establishing the status of the considered models (such as the LMSSM-6 presented here).
- There is an interesting complementarity between the supersymmetry spectrum such as from the LMSSM-6 global fit to data presented in this article, and similar spectra that could be constructed via the simplified models approach to supersymmetry phenomenology. With the global fit approach, the resulting spectra are guaranteed to be in agreement with the experimentally measured values of the observables used for the fitting procedure. This is not necessarily the case for simplified model spectra. It is interesting to explore this further and contrast this and similar characteristics.

## Footnotes

1 Throughout this article, we assume that it is possible to find a compactification with exactly the MSSM matter content. See [7–9] for instance and developments along this direction.

## References

[1] Douglas MR, Kachru S (2007). Rev. Mod. Phys..

[2] Denef F, Douglas MR, Kachru S (2007). Ann. Rev. Nucl. Part. Sci..

[3] Kachru S, Kallosh R, Linde AD, Trivedi SP (2003). Phys. Rev. D.

[4] Choi K, Falkowski A, Nilles HP, Olechowski M (2005). Nucl. Phys. B.

[5] Balasubramanian V, Berglund P, Conlon JP, Quevedo F (2005). JHEP.

[6] Conlon JP, Abdussalam SS, Quevedo F, Suruliz K (2007). JHEP.

[7] Braun V, He YH, Ovrut BA, Pantev T (2006). JHEP.

[8] M. Cicoli, S. Krippendorf, C. Mayrhofer, F. Quevedo, R. Valandro, JHEP **1307**, 150 (2013). doi:10.1007/JHEP07(2013)50. arXiv:1304.0022 [hep-th]

[9] Crispim Romão M, Karozas A, King SF, Leontaris GK, Meadowcroft AK (2016). Phys. Rev. D.

[10] AbdusSalam SS (2009). AIP Conf. Proc..

[11] AbdusSalam SS, Allanach BC, Quevedo F, Feroz F, Hobson M (2010). Phys. Rev. D.

[12] F. Quevedo. arXiv:1612.01569 [hep-th]

[13] S. Gukov, C. Vafa, E. Witten, Nucl. Phys. B **584**, 69 (2000). doi:10.1016/S0550-3213(01)00289-9, doi:10.1016/S0550-3213(00)00373-4. arXiv:hep-th/9906070. (Erratum: [Nucl. Phys. B **608** (2001) 477])

[14] Giddings SB, Kachru S, Polchinski J (2002). Phys. Rev. D.

[15] AbdusSalam SS, Conlon JP, Quevedo F, Suruliz K (2007). JHEP.

[16] Conlon JP, Kom CH, Suruliz K, Allanach BC, Quevedo F (2007). JHEP.

[17] Kallosh R, Wrase T (2014). JHEP.

[18] Bergshoeff EA, Dasgupta K, Kallosh R, Van Proeyen A, Wrase T (2015). JHEP.

[19] Kallosh R, Quevedo F, Uranga AM (2015). JHEP.

[20] Aparicio L, Quevedo F, Valandro R (2016). JHEP.

[21] Becker K, Becker M, Haack M, Louis J (2002). JHEP.

[22] M. Verzocchi in “talk at ICHEP 2008” (2008)

[23] ALEPH Collaboration, Phys. Rep. **427**, 257 (2006)

[24] R. Aaij et al. [LHCb Collaboration], Phys. Rev. Lett. **110**(2), 021801 (2013). doi:10.1103/PhysRevLett.1. arXiv:1211.2674 [hep-ex]

[25] A. Abulencia et al. [CDF Collaboration], Phys. Rev. Lett. **97**, 242003 (2006). doi:10.1103/PhysRevLett.97.242003. arXiv:hep-ex/0609040

[26] B. Aubert et al. [BaBar Collaboration], Phys. Rev. Lett. **95**, 041804 (2005). doi:10.1103/PhysRevLett.95.041804. arXiv:hep-ex/0407038

[27] E. Barberio et al. [Heavy Flavor Averaging Group]. arXiv:0808.1297 [hep-ex]

[28] E. Komatsu et al. [WMAP Collaboration], Astrophys. J. Suppl. **180**, 330 (2009). doi:10.1088/0067-0049/180/2/330. arXiv:0803.0547 [astro-ph]

[29] G. Aad et al. [ATLAS Collaboration], Phys. Lett. B **716**, 1 (2012). doi:10.1016/j.physletb.2012.08.020. arXiv:1207.7214 [hep-ex]

[30] S. Chatrchyan et al. [CMS Collaboration], Phys. Lett. B **716**, 30 (2012). doi:10.1016/j.physletb.2012.08.021. arXiv:1207.7235 [hep-ex]

[31] E. Barberio et al. [Heavy Flavor Averaging Group (HFAG)]. arXiv:0704.3575 [hep-ex]

[32] Feroz F, Hobson MP (2008). Mon. Not. Roy. Astron. Soc..

[33] F. Feroz, M.P. Hobson, M. Bridges, Mon. Not. Roy. Astron. Soc. **398**, 1601 (2009). doi:10.1111/j.1365-2966.2009.14548.x. arXiv:0809.3437 [astro-ph]

[34] J. Skilling, in *American Institute of Physics Conference Series*, ed. by R. Fischer, R. Preuss, U.V. Toussaint, pp. 395–405 (2004)

[35] Skands PZ (2004). JHEP.

[36] Allanach BC (2002). Comput. Phys. Commun..

[37] Belanger G, Boudjema F, Pukhov A, Semenov A (2009). Comput. Phys. Commun..

[38] Mahmoudi F (2008). Comput. Phys. Commun..

[39] Heinemeyer S, Hollik W, Stockinger D, Weber AM, Weiglein G (2006). JHEP.

[40] Heinemeyer S, Hollik W, Weber AM, Weiglein G (2008). JHEP.

[41] Dawson S, Eichten E, Quigg C (1985). Phys. Rev. D.

[42] Hewett JL, Lillie B, Masip M, Rizzo TG (2004). JHEP.

[43] G. Aad et al. [ATLAS Collaboration], JHEP **1510**, 054 (2015). doi:10.1007/JHEP10(2015)054. arXiv:1507.05525 [hep-ex]

[44] CMS Collaboration [CMS Collaboration], in *Proton–Proton Collisions at 13 TeV*. CMS-PAS-SUS-16-014

[45] CMS Collaboration [CMS Collaboration], CMS-PAS-SUS-16-015

[46] N. Nagata, H. Otono, S. Shirai. arXiv:1701.07664 [hep-ph]

